# N-acetyl-L-cysteine alleviated the oxidative stress-induced inflammation and necroptosis caused by excessive NiCl_2_ in primary spleen lymphocytes

**DOI:** 10.3389/fimmu.2023.1146645

**Published:** 2023-04-06

**Authors:** Xintong Zhang, Lihua Xu, Wenxue Ma, Bendong Shi, Qiaohan Liu, Yinghao Song, Cheng Fang, Pinnan Liu, Senqiu Qiao, Jingzeng Cai, Ziwei Zhang

**Affiliations:** ^1^ College of Veterinary Medicine, Northeast Agricultural University, Harbin, China; ^2^ Key Laboratory of the Provincial Education, Department of Heilongjiang for Common Animal Disease Prevention and Treatment, Northeast Agricultural University, Harbin, China

**Keywords:** NiCl2, oxidative stress, inflammation, necroptosis, mice spleen lymphocytes

## Abstract

**Introduction:**

Nickel (Ni) is widely used in industrial manufacturing and daily life due to its excellent physical and chemical properties. However, Ni has the potential to harm animals' immune system, and spleen is a typical immune organ. Therefore, it is crucial to understand the mechanism of NiCl_2_ damage to the spleen. The purpose of this study is to investigate the effects of different concentrations of NiCl_2_ exposure and intervening with strong antioxidants on spleen lymphocytes to better understand the damage mechanism of Ni on spleen lymphocytes.

**Methods:**

In this experiment, mice spleen lymphocytes were used as the research object. We first measured the degree of oxidative stress, inflammation, and necroptosis caused by different NiCl_2_ concentrations. Subsequently, we added the powerful antioxidant N-acetyl-L-cysteine (NAC) and used hydrogen peroxide (H_2_O_2_) as the positive control in subsequent experiments.

**Results:**

Our findings demonstrated that NiCl_2_ could cause spleen lymphocytes to produce a large number of reactive oxygen species (ROS), which reduced the mRNA level of antioxidant enzyme-related genes, the changes in GSH-PX, SOD, T-AOC, and MDA, the same to the mitochondrial membrane potential. ROS caused the body to produce an inflammatory response, which was manifested by tumor necrosis factor (TNF-α) in an immunofluorescence experiment, and the mRNA level of related inflammatory genes significantly increased. In the case of caspase 8 inhibition, TNF-α could cause the occurrence of necroptosis mediated by RIP1, RIP3, and MLKL. AO/EB revealed that spleen lymphocytes exposed to NiCl_2_ had significant necroptosis, and the mRNA and protein levels of RIP1, RIP3, and MLKL increased significantly. Moreover, the findings demonstrated that NAC acted as an antioxidant to reduce oxidative stress, inflammation, and necroptosis caused by NiCl_2_ exposure.

**Discussion:**

Our findings showed that NiCl_2_ could cause oxidative stress, inflammation, and necroptosis in mice spleen lymphocytes, which could be mitigated in part by NAC. The study provides a point of reference for understanding the toxicological effect of NiCl_2_. The study suggests that NAC may be useful in reducing the toxicological effect of NiCl_2_ on the immune system. The research may contribute to the development of effective measures to prevent and mitigate the toxicological effects of NiCl_2_ on the immune system.

## Introduction

Nickel (Ni) is a transition metal that is widely distributed in the atmosphere, water, and soil. However, due to its widespread industrial use, it has become a significant environmental pollutant. NiCl_2_, a compound containing nickel, is extremely toxic to both humans and laboratory animals (1). Studies have shown that exposure to NiCl_2_ is carcinogenic. NiCl_2_ have the potential to cause cancer through various mechanisms, including oxidative stress, genomic DNA damage, epigenetic changes, and activation of transcription factors (2). The carcinogenic effect of NiCl_2_ also included the induction of signal transduction and replication errors, which eventually led to cancer (3). NiCl_2_ exposure has been shown in studies to harm the reproductive system. NiCl_2_ could cross the placenta, causing placental malformation, as well as decrease placental viability, permeability changes, and potential embryotoxicity. A high dose of NiCl_2_ could cause sperm cell apoptosis and DNA damage, as well as induce testicular oxidative stress and generate a large number of reactive oxygen species (ROS). ROS had the potential to cause severe pathological damage and had an immediate impact on spermatogenesis (4), and N-acetyl-L-cysteine (NAC) is a known ROS scavenger that modulates ROS levels (5). Furthermore, studies had revealed that NiCl_2_ was immunotoxic. Knight et al. discovered that 0.5 mmol/g NiCl_2_ injection significantly reduced thymus weight, cortical lymphocytes showed obvious degenerative changes, and thymus reticular epithelium swelled and vacuolated (6). NiCl_2_ and its derivatives significantly reduced spleen index, and NiCl_2_ may also cause morphological changes in the spleen, resulting in a significant increase in the spleen’s red pulp area (7, 8). To investigate the mechanism of damage to the spleen by NiCl_2_, we isolated primary spleen lymphocytes from mice.

Cell death is a fundamental process that plays a role in various aspects of disease pathogenesis, including degenerative diseases, toxic diseases, inflammation, and tumors. Unlike apoptosis, necroptosis has been linked to the pathogenesis of many diseases (9). Necroptosis was an adjustable cell death pattern that resembled apoptosis. Necroptosis was classified into three types based on the driving factors: extrinsic necroptosis caused tumor necrosis factor (TNF-α), intrinsic necroptosis caused by ROS, and ischemia-mediated intrinsic necroptosis (10). A classic form of necroptosis, TNF-α mediated necroptosis results in the development of complex I, which includes TRADD, FADD, receptor-interacting serine/threonine protein kinase 1 (RIP1), and other proteins (11). Necroptosis was then brought about through the interaction of RIP1 and receptor-interacting serine/threonine protein kinase 3 (RIP3), as well as the phosphorylation of mixed lineage kinase domain-like protein (MLKL). According to studies, kinase inactive RIP1 expression or RIP3 or MLKL deletion prevented autoimmunity (12). Previous research had shown that cells undergoing necroptosis can activate the immune system, leading to tissue damage and inflammation. Furthermore, there is a significant association between necroptosis and heavy metal toxicity. Exposure to lead (Pb) was found to upregulate the expression of genes associated with both apoptosis and necroptosis in chicken models exposed to Pb or co-exposed to Pb and selenium. This effect was observed to be mediated by the activation of the MAPK signaling pathway. However, selenium was found to modulate this pathway, leading to a reduction in apoptosis and necroptosis of chicken kidney cells (13). Research has demonstrated that the inhibition of RIP1, RIP3, and MLKL expression by selenium yeast can effectively decrease cadmium (Cd) accumulation in the kidney and mitigate Cd-induced necroptosis (14). Additionally, previous studies have shown that particulate nickel and its derivatives cause more oxidative stress than water-soluble nickel compounds (15). Researchers have recently investigated the potential association between increased oxidative stress and impaired behavior as well as altered brain tissue morphology in rats exposed to subacute levels of nickel (16). Moreover, necroptosis is implicated in various inflammatory conditions. In mice, the knockout of RIP3 was found to prevent tissue damage caused by acute pancreatitis inflammation, thus inhibiting the development of pancreatitis (17). Mir-200a-5p could regulate necroptosis by targeting RNF11, which in turn regulates myocardial inflammation in chickens (18). Furthermore, experiments have demonstrated that increased oxidative stress plays a role in the induction of necroptosis in cardiomyocytes, resulting in left ventricular systolic dysfunction. Antioxidants have been found to potentially prevent heart failure by inhibiting necroptosis (19). Thus far, there has been limited discussion regarding the mechanism by which nickel induces immune damage.

Nickel (Ni) is a ductile and corrosion-resistant silver-white metal that has been widely utilized. While the toxicity of NiCl_2_ has been studied, the mechanism underlying its induction of necroptosis in lymphocytes of mice has received little attention. To elucidate the new mechanism underlying the cell injury induced by NiCl_2_, we evaluated the levels of oxidative stress, inflammation, and necroptosis in the lymphocytes of mice exposed to NiCl_2_. hydrogen peroxide (H_2_O_2_) was used as a positive control and NAC was added for intervention, and try to explain whether the toxicity of NiCl_2_ was affected after the oxidative stress was improved. This study will significantly enhance our understanding of the mechanisms underlying immune system damage in mice.

## Materials and methods

### Ethical statement and laboratory animals

Twenty-four male mice (average body weight 23 ± 2 g) were self-bred in our laboratory. In order to avoid unnecessary interference, mice freely ingested food and water and fed adaptively for 7 d before the experiment. All procedures were carried out following the directive of the Council of the European Communities (86/609/EEC) and approved by the Committee on Animal Management and Use of Northeast Agricultural University (SRM-11).

### Cell isolation and treatment

After decapitation, one mouse was immersed in bromogeramine and 75% alcohol. Then, we dissected the abdominal cavity of the mice and separated the spleen. The lymphocytes were separated using a mice spleen lymphocyte isolation solution kit (Beijing Solarbio Biological Manufacture CO., China). Cells were resuspended in RPMI+ medium (RPMI 1640 supplemented with 10% fetal bovine serum) and diluted to a density of 1 × 10^6^ cells/mL. According to previous studies and pre-experimental results, we decided to incubate lymphocytes with 0 (NC group),0.5 (0.5 mM group), 1.5 (1.5 mM group) and 2.5 (2.5 mM group) mM NiCl_2_ (purity 98%, Sigma, USA) for 18 h at 37°C in a humidified atmosphere with 5% CO_2_ (Set up 3 replicate wells for each concentration of each experiment). In order to explore the protective effect of NAC, we took 1.5mM NiCl_2_ as the research object, and set up control group (group NC), 150 μM NAC group (group NAC), 1.5 mM NiCl_2_ + 150 μM NAC group (group Ni+NAC), 1.5mM NiCl_2_ group (group Ni), and 100μM hydrogen peroxide group (group H_2_O_2_).

### Cell viability assay

Cell Counting Kit-8 (CCK-8 kit) was used to detect the survival rate of spleen lymphocytes under different drugs including NiCl_2_, H_2_O_2_ and NAC. The cells were cultured in 1640 medium containing different concentrations of NiCl_2_ (0mM,0.25mM,0.5mM,1.5mM,2.5mM), H_2_O_2_ (0 μM,50 μM,100 μM,150 μM,200 μM,500 μM), NAC (0 μM,50 μM,100 μM,150 μM,200 μM,500 μM) for18 h. The cell activity was detected by CCK-8 kit (Biosharp, China) and the OD value was measured by using a microplate reader (TECAN company, Switzerland) at 450 nm wavelength.

### ROS assay

ROS assay (Beyotime, China) for measuring ROS levels in mice spleen lymphocytes. Cells were incubated by medium with DCFH-DA fluorescent probe at 37 °C for 30 min (20). The cell were resuspended in PBS and observed under fluorescence microscope(Olympus Corporation, IX53, Japan). Image J software was used to quantify ROS-related fluorescence signals.

### Determination of MDA content and activity of GSH-PX, SOD, and T-AOC

The GSH-PX,MDA,SOD,and T-AOC kit (Nanjingjiancheng, China) were used to measure the antioxidant capacity of the spleen lymphocytes. The cells were collected by centrifugation, and then operated according to the manufacturer’s instructions.

### AO/EB staining

AO/EB staining was used to distinguish normal cell and necroptosis cells. were collected by centrifugation (1200 rpm, 5 min), rinsed with PBS, resuscitated and stained with AO/EB for 5 min. The morphology of the cells was observed by fluorescence microscope (Olympus Corporation, IX53, Japan).

### Detection of mitochondrial membrane potential

Mitochondrial membrane potential assay kit with JC-1 (Solarbio, China) was used to detect changes in mitochondrial membrane potential after exposure of spleen lymphocytes to NiCl_2_. Lymphocytes were added 0.5 mL JC-1 working solution for 20 min. Centrifuge at 4°C (1000 rpm, 5 min), discarded the supernatant, added JC-1 staining buffer to resuspended the cells, and then observed by fluorescence microscope (Olympus Corporation, IX53, Japan).

### Immunofluorescence

Immunofluorescence was used to detect the expression of TNF-α in spleen lymphocytes. Lymphocytes in each group were collected by centrifugation, resuspended in a 6-well plate with fixative, then alternately added primary antibody (proteintech, American) and secondary antibody (Abbkine, China) every 1 hour. After adding DAPI and standing for 5 minutes, the lymphocytes were observed under a fluorescence microscope (Olympus Corporation, IX53, Japan) (21).

### RNA isolation and real-time quantitative PCR analysis

Total RNA was separated from lymphocytes in the NC group and NiCl_2_ treatment groups by Trizol reagent at 18 h. cDNA was synthesized according to the instructions of the reverse transcription Kit (Transgene, Beijing, China). Specific primers (IL-8,TNF-α, iNOS, RIP1, RIP3, CPX,HO-1,CAT,SOD,IL-6,IL-1β, MLKL, FADD) for target genes (**Table 1**) were designed based on known sequences using Primer BLAST at the National Center for Biotechnology Information (NCBI). Quantitative real-time PCR (qPCR) was performed with a BIOER detection system (China, Hangzhou). Reactions were performed in a 10 μL reaction mixture containing 5 μL of 2 × SYBR Green I PCR Master Mix (R),1 μL of cDNA, 0.3 μL of each primer (10 μM) and 3.4 μL of PCR grade water.The relative abundance of each mRNA was calculated according to the 2^−ΔΔCt^ method and normalized to the mean expression of β-actin (22).

**Table 1:** The primers used in the present study.

IL-8	Forward 5'-TGTTCACAGGTGACTGCTCC-3'
	Reverse 5'-AGCCCATAGTGGAGTGGGAT-3'
TNF-α	Forward 5'-GATCGGTCCCCAAAGGGATG-3'
	Reverse 5'-CCACTTGGTGGTTTGTGAGTG-3'
iNOS	Forward 5'-AACTTGTTTGCAGGCGTCAG-3'
	Reverse 5'-CACATTGCTCAGGGGATGGA-3'
RIP1	Forward 5'-TCCTTAGAGGAGGACCAGCG-3'
	Reverse 5'-GGAGTTCGGTGCTGAAGTGG-3'
RIP3	Forward 5'-CCTCTCAGTCCACACTCCGA-3'
	Reverse 5'-CCGGGATTCCGTGACATAACT-3'
GPX	Forward 5'-TTGCTTCCACACCCCCATAC-3'
	Reverse 5'-TGCTGCACAGCAGGTGAATA-3'
HO-1	Forward 5'-GAAATCATCCCTTGCACGCC-3'
	Reverse 5'-CCTGAGAGGTCACCCAGGTA-3'
CAT	Forward 5'-AAGATTGCCTTCTCCGGGTG-3'
	Reverse 5'-TGTGGAGAATCGAACGGCAA-3'
SOD	Forward 5'-GCCCAAACCTATCGTGTCCA-3'
	Reverse 5'-AGGGAACCCTAAATGCTGCC-3'
IL-6	Forward 5'-GCCTTCTTGGGACTGATGCT-3'
	Reverse 5'-TGTGACTCCAGCTTATCTCTTGG-3'
IL-1β	Forward 5'-TGCCACCTTTTGACAGTGATG-3'
	Reverse 5'-TTCTTGTGACCCTGAGCGAC-3'
MLKL	Forward 5'-GCGTTGGCCCAAATTTGACT
	Reverse 5'-GGTCTTCTGCCTCGTTGACA-3'
FADD	Forward 5'-CCTTGGGGGAAGACACCATC-3'
	Reverse 5'-CAATGCGGAAGGCGATTGAG-3'

### Protein extraction and western blot analysis

Each group of cells were collected, lysed with RIPA and PMSF on ice, and then centrifuged at 4°C (12000 rpm for 25 min). SDS were added to the supernatant, boiled for 10 min, and preserved the samples at - 20°C for detection. The protein was isolated and transferred to nitrocellulose membrane by SDS- polyacrylamide gel electrophoresis, where the control lanes were loaded side-by-side with the lanes for the proteins of interest, and the membranes were then vertically cut. Immunoblotting was then performed by blocking with 5% skimmed milk at 37°C for 2h and incubating with diluted primary antibody overnight (23). Detection of protein expression of necroptosis related signaling pathway (RIP1, RIP3, MLKL, Caspase 8, NLRP3), The dilution ratio of primary antibody were shown in **Table 2**. Then, the membranes were washed three times with TBST, incubated with secondary antibody at room temperature for 1h, and then washed three times with TBST again. The protein bands were examined using enhanced chemiluminescence detection reagents (Applygen Technologies Inc., Beijing, China) and X-ray films (TransGen Biotech Co., Beijing, China). The relative abundance of proteins was standardized as β-actin (24).

**Table 2:** The antibodies used in the present study.

Antibody name	Dilution ratio	Company information
RIP1	1:800	Proteintech,, China
RIP3	1:15000	Proteintech, China
MLKL	1:1000	Proteintech, China
Caspase 8	1:1000	Cell Signaling Technology, Inc., USA
TNF-α	1:1000	Wanlei,China
β-actin	1:10000	ABclonal, China
NLRP3	1:10000	Abmart, China
Second Antibody	1:10000	Proteintech, China

### Data analysis

Statistical analysis of all data was carried out with one-way analysis of variance (ANOVA) using GraphPad Prism Version 8.0 software, and Tukey’s Multiple Comparison Test was used to analyze the differences. All the experiments were performed at least six times. The results were expressed as the means ± standard deviations (SD), ns represents no statistical difference between the two groups, * represents significant difference between the two groups (P<0.05), and ** represents extremely significant difference between the two groups (P<0.01).

## Results

### NiCl_2_ induced oxidative stress in spleen lymphocytes

**Figure 1:**
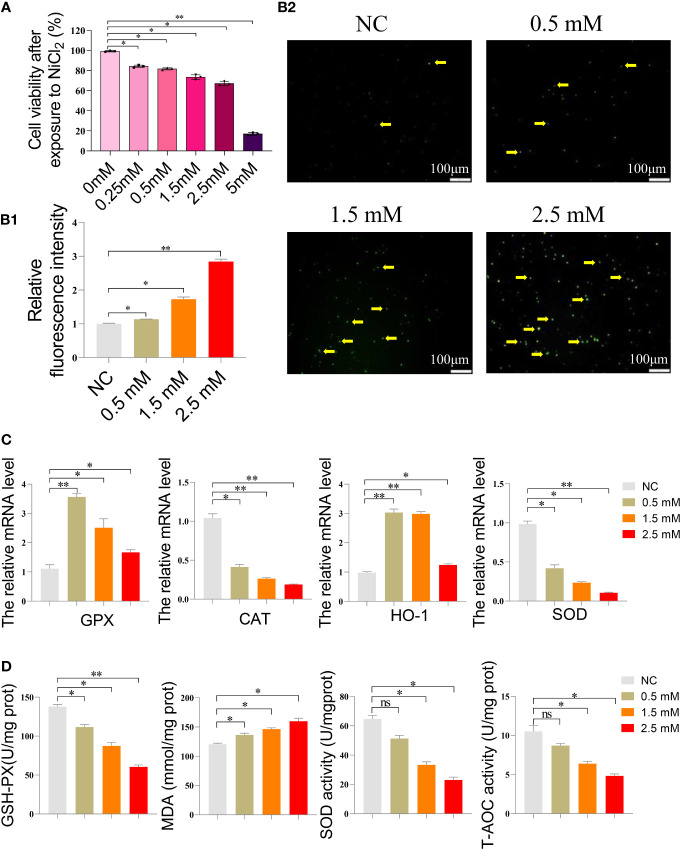
**(A)** Cell survival rate of spleen lymphocytes exposed to different concentrations of NiCl_2_. **(B)** Reactive oxygen species (ROS) staining results of spleen lymphocytes exposed to different concentrations of NiCl_2_, The green dot pointed by the yellow arrow represents the ROS level. ImageJ analysis of the mean fluorescence intensity of ROS staining in each group. * represents P<0.05 compared with the NC group, and ** represents P<0.01 compared with the NC group. **(C)** Expression of antioxidant enzyme-related gene mRNA of spleen lymphocytes exposed to different concentrations of NiCl_2_. ns represents no statistical difference between the two groups, * represents significant difference between the two groups (P<0.05), and ** represents extremely significant difference between the two groups (P<0.01). **(D)** After exposed to different concentrations of NiCl2, the content of GSH-Px and MDA, as well as the activity of SOD and T-AOC, were measured in spleen lymphocytes. ns represents no statistical difference between the two groups, * represents significant difference between the two groups (P<0.05), and ** represents extremely significant difference between the two groups (P<0.01). We have uploaded the revised file.

In **Figure 1A**, a decrease in cell viability was observed in spleen lymphocytes exposed to increasing concentrations of NiCl_2_ for 18 hours. **Figure 1B** depicts the ROS levels in the cells, where green fluorescent dots indicate ROS levels. Spleen lymphocytes exposed to NiCl_2_ exhibited a significant increase in ROS levels compared to the NC group (P <0.05). **Figure 1C** displays the results obtained from qRT-PCR analysis of the mRNA levels of CAT, SOD, GPX, and HO-1 in spleen lymphocytes. The results indicate a decrease in the mRNA levels of these genes with increasing concentrations of NiCl_2_ when compared to the NC group. In **Figure 1D**, it can be observed that the activity of T-AOC and SOD, as well as the content of GSH-PX, were significantly decreased (P < 0.05) following treatment with NiCl_2_. Conversely, the content of MDA showed a significant increase (P < 0.05).

### NiCl_2_ reduced the mitochondrial membrane potential and induced inflammation in spleen lymphocytes

**Figure 2:**
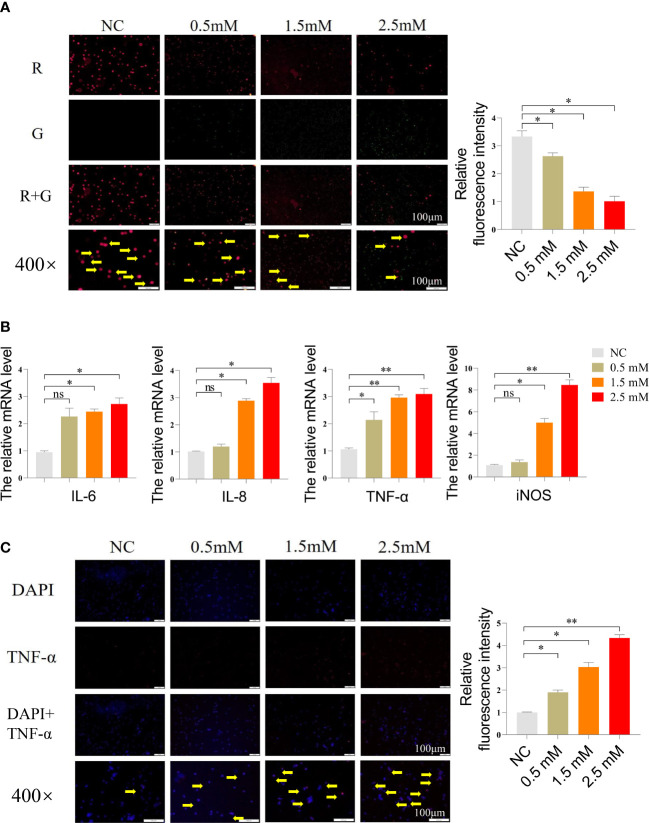
**(A)** Mitochondrial membrane potential detection of spleen lymphocytes exposed to different concentrations of NiCl_2_, the transition from red dots (pointed by the yellow arrow) to green dots indicates a decrease in mitochondrial membrane potential. **(B)** The mRNA expression of inflammation-related genes in spleen lymphocytes exposed to different concentrations of NiCl_2_, ns represents no statistical difference between the two groups, * represents significant difference between the two groups (P<0.05), and ** represents extremely significant difference between the two groups (P<0.01). **(C)** Immunofluorescence detection of TNF-α in spleen lymphocytes exposed to different concentrations of NiCl_2_, blue dots represent cell nucleus, red dots represent the expression of TNF-α.

Next, we measured the mitochondrial membrane potential of spleen lymphocytes using JC-1 working solution, which emits red fluorescence when the mitochondrial membrane potential is high and green fluorescence when it is low. The findings are presented in **Figure 2A**, which showed that the NC group exhibited the highest red fluorescence intensity, while the remaining three groups displayed varying levels of green fluorescence. Notably, the highest level of green fluorescence was observed at a NiCl_2_ concentration of 2.5mM. Furthermore, we conducted additional analyses to assess the mRNA expression levels of inflammation-related genes, such as IL-6, IL-8, TNF-α, and iNOS. **Figure 2B** illustrated that the mRNA expression levels of inflammation-related genes, namely IL-6, IL-8, TNF-α, and iNOS, were significantly elevated in spleen lymphocytes following NiCl_2_ exposure (P < 0.05). Furthermore, the levels of these genes exhibited a positive correlation with the NiCl_2_ concentration. Notably, at a concentration of 2.5 mM, the observed enhancement was more pronounced than that in the NC group (P < 0.01). In addition, the immunofluorescence results, presented in **Figure 2C**, showed a gradual increase in the expression of TNF-α with the increasing NiCl_2_ concentration, which was consistent with the mRNA expression levels observed earlier.

### NiCl_2_ induced necroptosis in spleen lymphocytes

**Figure 3:**
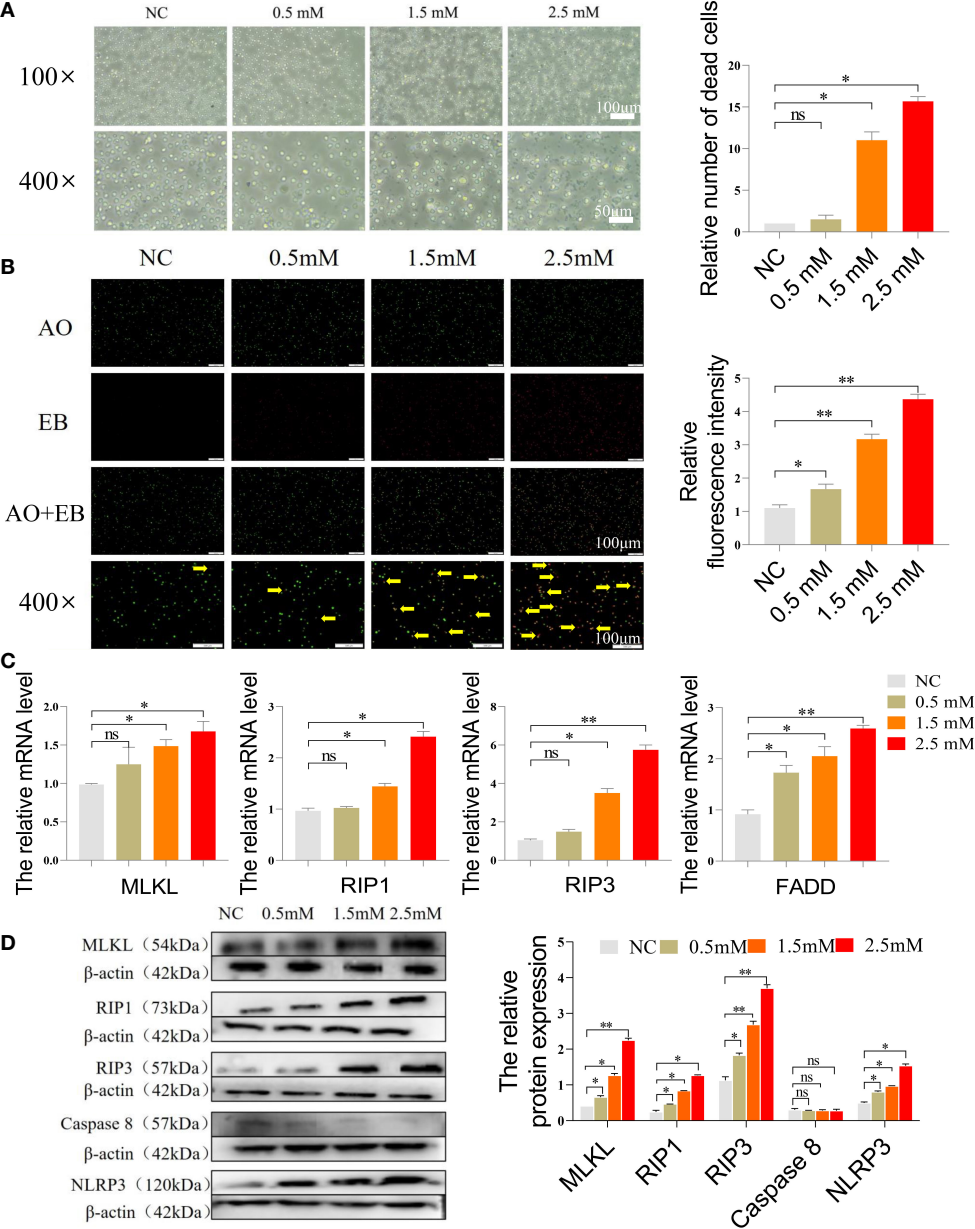
**(A)** Microscopic morphology of cells after exposure to different concentrations of NiCl_2_. **(B)** AO/EB staining of spleen lymphocytes exposed to different concentrations of NiCl_2_, green dots represent normal cells and red dots pointed by the yellow arrow represent necroptosis cells. **(C)** The mRNA expression of necroptosis-related genes in spleen lymphocytes exposed to different concentrations of NiCl_2_. ns represents no statistical difference between the two groups, * represents significant difference between the two groups (P<0.05), and ** represents extremely significant difference between the two groups (P<0.01). **(D)** The protein expression of necroptosis-related genes in spleen lymphocytes exposed to different concentrations of NiCl_2_.

**Figure 3A** displayed the morphology of the cells under the microscope after they were exposed to different concentrations of NiCl_2_. As the concentration increased, there was more and more cellular debris in the field of view. **Figure 3B** presented the results of the AO/EB stain of spleen lymphocytes. In this stain, red cells represent necroptotic cells, orange cells represent apoptotic cells, and green cells represent normal cells. Our findings indicated that the NC group exhibited the highest number of green normal cells and the lowest number of red necroptotic cells among the four groups. However, with the gradual increase of NiCl_2_ concentration, the number of red necroptotic cells increased significantly. We further detected the mRNA expression of necroptosis related genes, **Figure 3C** demonstrated that the mRNA expression levels of MLKL, RIP1, RIP3, and FADD were significantly higher than those observed in the NC group (P < 0.05), particularly in the group exposed to 2.5 mM NiCl_2_. Additionally, Western blotting analysis revealed increased protein expression of MLKL, RIP1, RIP3, and NLRP3 in the other three NiCl_2_ exposure groups when compared to the NC group (**Figure 3D**).

**Figure 4:**
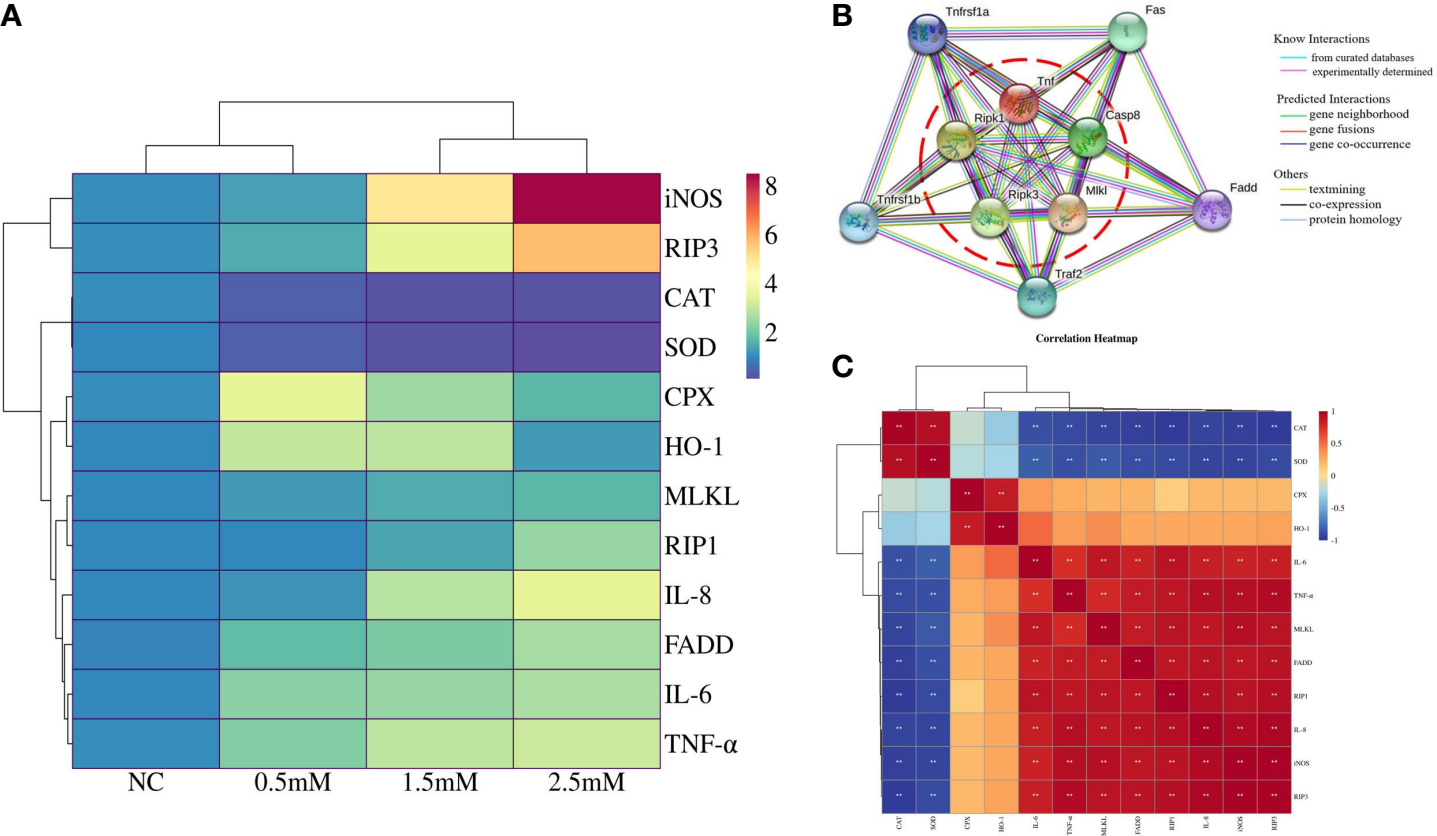
**(A)** Correlation heatmap indicates correlation of detected gene expression in spleen lymphocytes exposed to different concentrations of NiCl_2_, red indicates positive correlation and blue indicates negative correlation. **(B)** Protein-protein interaction (PPI) network regulated between antioxidative enzymes genes, inflammation, and necroptosis based on the String database in spleen lymphocytes exposed to different concentrations of NiCl_2_. **(C)** Heatmap of cluster analysis in spleen lymphocytes exposed to different concentrations of NiCl_2_. Blue represents downregulated while red represents upregulated.

**Figure 4A** indicated correlation of detected gene expression in spleen lymphocytes exposed to different concentrations of NiCl_2_, antioxidative enzyme-related genes were negatively correlated with inflammation and necroptosis, while inflammation and necroptosis-related genes were positively correlated. Protein-protein interaction (PPI) network showed the association between genes (**Figure 4B**), the interaction proteins included RIP1, RIP3, MLKL, TNF-α.We used heat maps to analyze the average of the quantized results for each marker in each group, as shown in **Figure 4C**.

### NAC mitigated the development of oxidative stress, inflammation, and necroptosis induced by NiCl_2_

**Figure 5:**
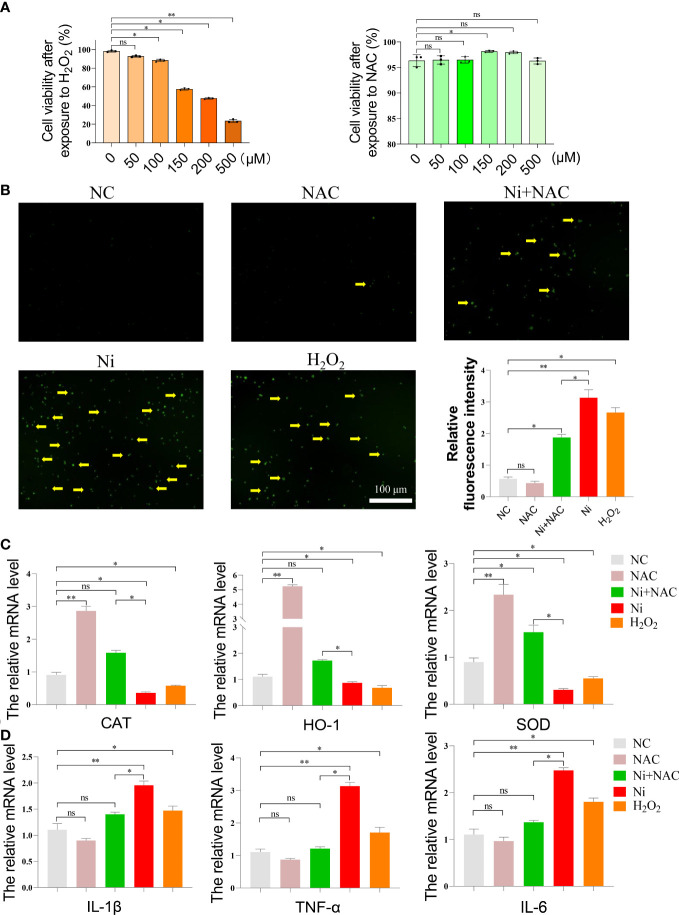
**(A)** Cell survival rate of spleen lymphocytes exposed to different concentrations of N-acetyl-L-cysteine (NAC) and hydrogen peroxide (H_2_O_2_). **(B)** ROS staining of spleen lymphocytes exposed to NiCl_2_, H_2_O_2_ and NAC. The green dot pointed by the yellow arrow represents the ROS level. ImageJ analyzes the average fluorescence intensity of ROS staining in each group. ns represents no statistical difference between the two groups, * represents significant difference between the two groups (P<0.05), and ** represents extremely significant difference between the two groups (P<0.01). **(C)** mRNA expression of antioxidant enzyme-related genes after spleen lymphocytes exposed to NiCl_2_, H_2_O_2_ and NAC. **(D)** mRNA expression of inflammatory-related genes after spleen lymphocytes exposed to NiCl_2_, H_2_O_2_ and NAC.

After NiCl_2_ exposure, NAC was added to spleen lymphocytes as a potent antioxidant, and H_2_O_2_ was used as a positive control. The survival rates of spleen lymphocytes after exposure to various concentrations of NAC and H_2_O_2_ were shown in **Figure 5A**. The ROS levels in each group were presented in **Figure 5B**, with the NC and NAC groups exhibiting lower ROS levels, while the Ni and H_2_O_2_ groups displayed higher ROS levels. Notably, the ROS level in the Ni+NAC group was significantly lower than that in the Ni group.

In order to verify the protective effect of NAC on spleen lymphocytes after NiCl_2_ exposure, we further detected the genes related to oxidative stress(CAT,SOD,HO-1), inflammation(IL-1β,TNF-α,IL-6) and necroptosis(RIP1,RIP3,MLKL). The results were shown in the **Figures 5C**, **D**. Firstly, **Figure 5C** showed that compared with the NC group, the mRNA expression of CAT,SOD,HO-1 decreased significantly in Ni group(P <0.05), and the mRNA expression of CAT,SOD,HO-1 in Ni+NAC group was higher than that in Ni group(P <0.05).As shown in **Figure 5D**, compared with the NC group, the mRNA levels of inflammatory factors(IL-1β,TNF-α,IL-6) were significantly increased after NiCl_2_ exposure. In addition, the mRNA levels of inflammatory factors(IL-1β,TNF-α) in the Ni+NAC group were lower than those in the Ni group.

**Figure 6:**
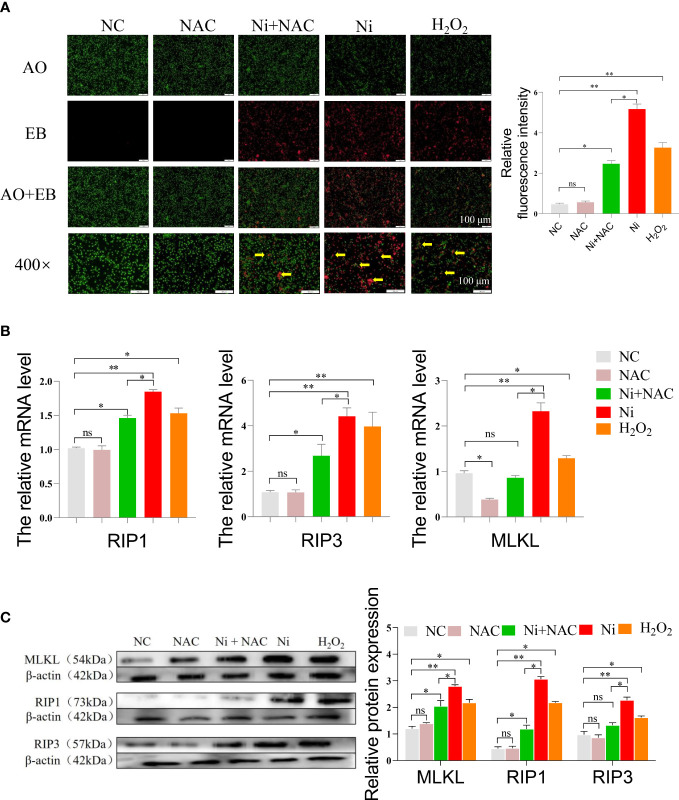
**(A)** AO/EB staining of spleen lymphocytes after exposure to, green dots indicate normal cells, and red dots pointed by the yellow arrow indicate necroptosis cells. **(B)** The mRNA expression of necroptosis-related genes in spleen lymphocytes exposed to NiCl_2_, H_2_O_2_ and NAC. ns represents no statistical difference between the two groups, * represents significant difference between the two groups (P<0.05), and ** represents extremely significant difference between the two groups (P<0.01). **(C)** The protein expression of necroptosis-related genes in spleen lymphocytes exposed to NiCl_2_, H_2_O_2_ and NAC.

After AO/EB staining, only a few necroptotic cells were observed in the NC and NAC groups. The number of necroptotic cells in the Ni group and H_2_O_2_ group was higher compared to the NC group. The necroptosis level in spleen lymphocytes of the Ni+NAC group was significantly reduced when compared to the Ni group, however, it was still higher than that in the NC and NAC groups (**Figure 6A**).

We conducted an analysis of the mRNA expression levels of necroptosis-related genes, specifically RIP1, RIP3, and MLKL. **Figure 6B** showed that the mRNA levels of these genes were higher in the Ni group compared to the NC and Ni+NAC groups. To further investigate the necroptosis pathway, we performed Western blot analysis on key proteins involved in the pathway. **Figure 6C** demonstrated that the protein expression of RIP1, RIP3, and MLKL was consistent with the RT-PCR results. After NiCl_2_ treatment, the protein expression of RIP1, RIP3, and MLKL in spleen lymphocytes significantly increased (P<0.05). However, compared to the Ni group, the protein expression of RIP1, RIP3, and MLKL decreased in the Ni+NAC group (P<0.05).

## Discussion

Studies have shown that excessive exposure to heavy metals can cause damage to the body. For example, Pb could cause oxidative stress and inflammation in the liver, and produce severe neurological damage (25, 26). A growing body of literature recognizes that NiCl_2_-induced toxicity may also be mediated by ROS (27). ROS is the most common type of free radical found in organisms (28). ROS was maintained by the cellular processes that produced ROS and antioxidant defense (29). Oxidative stress described the damage that occurs when ROS overcame the antioxidant defense system, it occurs when the production of ROS becomes too much or the internal balance was destroyed due to the reduction of defense system (30). It was well established from a variety of studies, that oxidative stress could disrupt a variety of signal pathways and affect a variety of biological processes by modifying proteins, promoting inflammation, inducing apoptosis, relieving autophagy, damaging mitochondrial function and other mechanisms (31). In this study, NiCl_2_ was found to cause the level of ROS increased significantly with the gradual increase of NiCl_2_ concentration, and the mRNA levels of antioxidant enzyme related genes (CAT, SOD, CPX and HO-1) decreased in varying degrees, suggesting that spleen lymphocyte exposed to NiCl_2_ had obvious oxidative stress. NAC was a powerful antioxidant. It had the function of scavenging free radicals, scavenging ROS and inhibiting the formation of ROS. In this study, the use of NAC significantly reduced the level of ROS, and the mRNA levels of CAT, SOD and HO-1 were significantly higher than those in Ni group. These results suggested that the oxidative stress caused by NiCl_2_ could be partially alleviated by NAC. In addition, because oxidative stress could affect mitochondria, we also detected the mitochondrial membrane potential of spleen lymphocytes. The mitochondrial membrane potential decreased after exposure to NiCl_2_, suggesting an abnormal state of mitochondria due to NiCl_2_.

Previous research has that oxidation and inflammation were closely related in the process of maintaining the body’s immune response (32). At physiological concentrations, ROS could act as a second messenger in cellular processes such as inflammation, cell growth and differentiation (33). Oxygen free radical was the effector of inflammatory response, and the excessive production of oxygen free radical could also induce inflammatory response (34–36). It had previously been observed that NiCl_2_-mediated reductions in antioxidant enzyme activity and increases in TNF-α and IL-1β levels were significantly abrogated by zinc in rat brain and liver (37). Study have demonstrated that Equisetum hyemale Linnaeus can mitigate the elevation of TNF-α, IL-6, and IL-8, thereby reducing the inflammatory response (38). It had previously been observed that curcumin has the ability to modulate inflammatory cytokines such as IL-6, IL-8, and TNF-α, which can help alleviate inflammation (39). According to this study, it was discovered through immunofluorescence that the exposure to NiCl_2_ caused a significant increase in the expression of TNF-α in spleen lymphocytes. At the same time, the experiments showed that the mRNA levels of IL-6, IL-8, TNF-α, and iNOS also significantly increased after the exposure to NiCl_2_, our research was consistent with previous theories (40). After the use of NAC, we found that not only the mRNA levels of antioxidant enzymes were increased, the expression of ROS was down-regulated, but also the mRNA levels of inflammatory factors were also decreased, indicating that with the weakening of oxidative stress, the inflammatory response was also weakened accordingly. However, studies have shown that when the concentration of nickel chloride is 3.3mg/kg, NiCl_2_ inhibits the production of TNF-α, and suppresses T-cell function and promotes an immunesuppressive macroscopical phenotype in rats (41). According to this study and our experimental results, it is not difficult to conclude that low-dose NiCl_2_ could protect the immune system by down-regulating TNF-α, and excessive nickel chloride will inevitably damage the immune system. It is noteworthy that TNF-α plays an important role in the biological effects of NiCl_2_ at any concentration.

TNF-α was reported to induce apoptosis and a form of cell death that exhibits morphological characteristics of necroptosis. Studies on this dual role showed that TNF-α was required to induce necroptosis under the action of RIP1, RIP3 and MLKL when caspase 8 was inhibited (42). In this study, NiCl_2_ were found to cause necroptosis accompanied by massive ROS production and inflammation. The mRNA and protein levels of necroptosis-related genes (RIP1, RIP3, MLKL) increased with the increase of NiCl_2_ concentration, but decreased under the condition of weakened oxidative stress and inflammation.

## Conclusion

Nowadays, the compounds of Ni had been widely used which excessive exposure could cause damage to humans and animals. The present study was designed to determine the effect of NiCl_2_ on spleen lymphocyte, and the results showed that NiCl_2_ leads to necroptosis by causing high expression of oxidative stress and inflammatory-related factors in mice spleen lymphocytes, this situation was partially improved after the use of the antioxidant NAC. This experiment provided a reference for understanding the damage mechanism and protection of excessive NiCl_2_ on the immune system.

## Data availability statement

The raw data supporting the conclusions of this article will be made available by the authors, without undue reservation.

## Ethics statement

The animal study was reviewed and approved by All procedures were carried out following the directive of the Council of the European Communities (86/609/EEC) and approved by the Committee on Animal Management and Use of Northeast Agricultural University (SRM-11).

## Author contributions

XZ: Conceptualization, Methodology, Writing – original draft. LX: Conceptualization, Validation, Data curation. WM and JC: Investigation, Formal analysis. BS and QL: Writing – review and editing. CF and PL: Software, Methodology. SQ and YS: Investigation, Visualization. ZZ: Supervision, Writing – review and editing, Funding acquisition. All authors contributed to the article and approved the submitted version.
